# Evidence‐based facilitator strategies for enhancing social engagement in groups of older adults with ADRD

**DOI:** 10.1002/alz.70131

**Published:** 2025-04-10

**Authors:** Joseph McKinley, Alice W. Brumley, Christine L. Williams, Emmanuelle Tognoli, Christopher Beetle

**Affiliations:** ^1^ Department of Physics Florida Atlantic University Boca Raton Florida USA; ^2^ Center for Complex Systems and Brain Sciences Florida Atlantic University Boca Raton Florida USA; ^3^ College of Nursing Florida Atlantic University Boca Raton Florida USA

**Keywords:** Alzheimer's disease and related dementias, complex systems, group interventions, group leadership, interdisciplinary research, social engagement, social groups, social isolation, virtual socialization

## Abstract

**INTRODUCTION:**

Alzheimer's disease and related dementias (ADRD) is exacerbated by social isolation. One potential intervention for improving the health outcomes of individuals with ADRD is by providing opportunities for socialization that are highly engaging.

**METHODS:**

To identify strategies for enhancing social engagement, we studied recorded virtual group sessions of older adults aged ≥ 65, with mild to moderate ADRD who interacted with younger adult facilitators. We developed multivariate regression models that use data describing group behavior, activity, and composition to predict enhanced engagement.

**RESULTS:**

We identified predictors of enhanced engagement related to group composition, structure, leadership, and mode of delivery. These results inform strategies for designing group settings which maximize engagement by using the synergistic influence of whole social groups on individuals.

**DISCUSSION:**

We make evidence‐based treatment recommendations for facilitators seeking to maximize engagement and make recommendations for future research investigating the preservation or loss of social engagement.

**Highlights:**

- Social isolation is a major contributing factor to the development of Alzheimer's disease and related dementias.
- Virtual socialization can help mitigate lack of opportunity for social contact.
- Audio–video data describing group activity were used to predict social engagement.
- Group size, behavior, composition, and time spoken contributed to social engagement.
- Experienced group facilitators maximized engagement by supporting conversation.

## 1 BACKGROUND

While the global incidence of Alzheimer's disease and related dementias (ADRD) is increasing, currently there is no cure.^1^ More than 55 million people live with ADRD, creating a critical need to mitigate the impact on families and health services. One factor that increases the risk of ADRD is low social contact in later life.^2^, ^3^, ^4^, ^5^ Smaller social networks and decreased social connections exacerbate cognitive decline. More broadly, reduced levels of social engagement are associated with premature mortality. In an 18 year longitudinal study, persistent loneliness in mid‐life, but not intermittent loneliness, was associated with later development of ADRD in individuals without high genetic risk, that is, apolipoprotein E ε4 non‐carriers.^6^

Recent studies have also linked anatomical brain changes and cognitive dysfunction to decreased social engagement, supporting a bidirectional relationship. Reduced brain volume is associated with loneliness and isolation.^7^ Reduced cognitive stimulation, insufficient emotional support, and subsequent depression accompanying social isolation may explain the relationships among social disengagement, loneliness, and cognitive decline.^8^ Cognitive dysfunction from ADRD‐related brain changes in particular decreases social engagement, further exacerbating social isolation and exclusion.^9^ Consequently, social interventions for older adults at risk for cognitive decline, including those affected by ADRD, are essential.

To guide professionals seeking effective interventions, we developed a systemic, multidimensional approach that compared various data describing different aspects of social groups and determined the relationship of these data with the observed levels of engagement in these groups. Our work is informed by the theoretical analysis of complex systems. A key property observed in complex systems composed of many (i.e., more than two) interacting agents is the formation of spatiotemporal patterns in which the dynamics of the whole system are not reducible to the pairwise interactions of its subsystems. Accordingly, we expect the social behavior of a group of three or more interacting agents to be fundamentally more complex and have richer engagement than that of a pair of interacting agents.^10^, ^11^, ^12^ Many complex systems also exhibit the formation of collective patterns of behavior among their constituent parts, a phenomenon called entrainment.^13^, ^14^, ^15^, ^16^ Complexity science offers tools for quantifying individual and collective behavior,^17^, ^18^, ^19^ accounting for systemic whole‐group effects on individuals and identifying cues to recognize when such effects are important. This wholistic perspective offers new insights for describing and predicting how the behavior of individuals is altered by their embedding in larger social networks, and reflects and complements other emerging research in the study of healthy aging.^20^

To date, there is little research providing evidence‐based recommendations for group facilitators interested in maximizing social engagement among older adults with ADRD. Zagic et al.^21^ conducted a systematic review of randomized controlled trials of interventions for social isolation in adults including group interventions with mean age > 65 but none included individuals with ADRD. Self‐reported social contact improved in group formats, but perceived quality of social connections did not necessarily follow. Li et al.^22^ conducted a meta‐analysis of interventions to reduce social isolation among older adults during the COVID‐19 pandemic. Interventions that improved self‐esteem and encouraged self‐expression had positive effects, but the target population did not include older adults with ADRD.

We investigated recordings of virtual social interactions of older adults with ADRD and younger unimpaired adult facilitators using concepts from complexity science including entrainment, systemic whole‐group effects, and third‐party stimulation. Our analysis combined qualitative and quantitative data measuring group composition and dynamic activity in multivariate regression models predicting the observed engagement quality over time and identified predictors of enhanced engagement. This work fills a gap in the literature by combining the interdisciplinary approaches of data, complexity, and nursing science toward the common goal of identifying group intervention strategies for improving the social and cognitive health of older adults with ADRD. We make evidence‐based recommendations for facilitators to design group settings which maximize engagement, thus reducing isolation and improving the older adults’ health.

## 2 METHODS

### 2.1 Study overview

This study was conducted by an interdisciplinary team of applied geropsychiatric nurse scientists and fundamental research scientists to gain an understanding of the principles underlying preservation or loss of social interaction in aging and in ADRD‐related cognitive decline. We examined evidence of enhanced social engagement in moderated group discussions where professional and student facilitators conducted virtual group sessions with older adults attending a day program at a memory and wellness center (MWC) for those affected by ADRD.

### 2.2 Sample and setting

In 2020, the COVID‐19 pandemic severely disrupted regular social interactions for many vulnerable older adults, including our study participants. These included individuals attending an adult day program for people with mild to moderate ADRD at a university‐affiliated MWC. The MWC is a nurse‐run, on‐campus facility that offers diagnostic and therapeutic services for individuals with memory disorders and their family caregivers, as well as educational opportunities for students from a variety of disciplines. The MWC is located in a coastal, urban setting that is predominantly White, non‐Hispanic, 59.6% college educated, with a median annual household income > $95,000. Although there are costs associated with attending the day program, scholarships are available to waive those costs for individuals unable to afford them. Participant sociodemographic characteristics reflected the community. The study was originally designed for in‐person observations at the MWC, but the pandemic made it necessary to modify the data collection plan when in‐person activities were suspended to refocus on observations within recorded online group sessions held using the video conferencing software WebEx.

Day program attendees were deeply affected by the COVID‐19–related suspension of in‐person activities, which previously had provided vital opportunities for socialization and cognitive enrichment. Approximately 100 enrollees were left with limited opportunities for socialization outside the home. To respond, staff developed virtual activities as temporary substitutes for in‐person contact. Staff and family members assisted enrollees to join online sessions held several times per week. Activities included group games and discussions tailored to participants’ ability and preferences. Each of the sessions consisted of a variety of speech‐related activities, including question and answer–based trivia games such as Jeopardy and Who Wants to be a Millionaire, Boggle and other spelling‐based games, and games in which participants were asked to name the concluding words of common phrases. Activities also included games in which participants were asked to sing the missing lyrics of songs and discussions involving complex social issues such as asking participants for their opinions regarding fictitious court cases or celebrities. In addition, there were spontaneous departures from the games and prescribed topics, for example, when the younger control subject showed her pet cat to the camera and lengthy discussions around pets followed.

To maximize diversity, equity, and inclusion, all individuals regardless of culture, race, sex, gender, or sexual orientation who attended the MWC day program were eligible to be part of the study. There were no costs to participants for attendance at the virtual sessions available during the study period. A total of 16 older adult day center attendees were recruited. In addition, four cognitively healthy adult hosts and one graduate student research assistant who occasionally engaged in conversations with the rest of the group participated. The study sample consisted of nine females and seven males aged ≥ 65, and one control (a younger adult female) who attended 30 minute virtual sessions over a 9 month period, with the first session used in this study dated September 4, 2021 and the last session dated November 11, 2021. By watching the videos, we were able to observe the participants’ preferred gender pronouns, which identified the participants as cisgender (i.e., the nine females identified as women, and the seven males identified as men). This was also the case for the hosts and the control subject. Each video (*N* = 30) was ≈ 30 minutes in length, so a total of ≈ 900 minutes or 15 hours of audio–video data were collected, analyzed, and watched by trained professionals.

**RESEARCH IN CONTEXT**

1. **Systematic review**: Our review of the literature (PubMed, APA PsychNet, PsychInfo) identified social isolation as a growing health crisis and significant contributing factor to the development and worsening of Alzheimer's disease and related dementias (ADRD). Scant research has addressed enhancing the quality of social interactions among groups of individuals with ADRD as a health intervention.
2. **Interpretation**: Using mixed methods, we identified factors that contribute to enhanced social engagement in group settings and the utility of virtual interactions for improving health by maintaining socialization for otherwise isolated individuals. Using multivariate regression, our team identified strategies that facilitators can use to engineer group activities to maximize social engagement in groups of older adults with ADRD.
3. **Future directions**: Future research should examine the efficacy of virtual group engagement to improve the health of individuals with ADRD. We identified group structures, facilitator strategies, and modes of delivery that may enhance social engagement in future studies.

All participants with mild to moderate cognitive impairment due to ADRD, who were enrolled in the MWC day center program at the time of the study, and who had a family member who gave consent and agreed to be available to assist the participant to access the online sessions, were eligible for the study. There was no requirement for participants to have prior experience with virtual socialization software technology, and those who struggled to use the technology were assisted by their family members when needed. There were no exclusion criteria.

The number of MWC participants ranged from two to nine participants per session and most attended at least one of the two sessions offered per week. The control attended all but two sessions. Thirty sessions were selected for analysis based on the quality of the recording and interactions. Sessions were led by one of four facilitators (Hosts), one of whom was a professional MWC staff member, while the others were students who were trained and supervised by a staff expert and a gerontological nurse graduate research assistant. MWC staff invited day program attendees that had a family member or aide who could assist with signing into WebEx. Family members were contacted by MWC staff and gave verbal consent for the research. The research team created a video introduction of the research, which was shown before new participants joined WebEx sessions. MWC attendees assented to participate virtually in the presence of staff, family members, and a trained nursing research assistant. The study was approved by the university institutional review board and conducted according to the ethical standards as laid down by the 1964 Declaration of Helsinki and its subsequent amendments. Participants met the inclusion criteria as currently enrolled in the MWC day center program.

### 2.3 Design

This mixed‐method study incorporated qualitative and quantitative approaches. Qualitative and quantitative data were first analyzed separately and then combined to determine their relative impact on participants’ social engagement. These data, which are described in more detail in the following subsections, included acoustic–prosodic data such as measures of speaker pitch and intensity, time‐based data such as the amount of time spoken by the various speaker groups, binary‐coded variables identifying instances of particular behaviors like use of humor or some notable non‐verbal responses, the number of people speaking at a particular moment in time, the identity of the host for each session, and subjective ratings of the level of engagement as determined by investigators.

### 2.4 Event coding and behavioral‐group analyses

To analyze the social interactions described previously, our team developed a method for coding qualitative behavioral variables to identify behavioral indicators relevant to social engagement. This coding process consisted of identifying time segments in the videos, referred to as events, in which some notable, self‐contained interaction between various group members occurred. In general, a single event contained multiple speech segments corresponding to different speaker turns. Events were identified for each of the videos, with one video being marked as having as few as six events (see Table 1) and one as many as 209, and most falling somewhere in between, with a total of 1839 events across the 30 videos (where each event has 17 features in total, with the engagement score as the response, discussed in detail in the following subsections). Typically, an event began when the facilitator asked a question or introduced a topic and ended after the last response to the topic. To reduce bias, two professionally trained investigators worked together to identify events, producing one common set of data.

**TABLE 1:** An example of the social engagement event data developed by our team to identify relevant factors for enhancing engagement (# = number speaking, score = engagement score).

Topic	Start time	End time	Social	Humor	Game	Cue	Affirm	Disc.	Correct	NVerb	#	Host 1	Host 2	Host 3	Score
Boggle intro	0 s	36 s	0	0	1	1	0	0	0	0	1	1	0	0	1
Boggle set up	37 s	80 s	0	0	1	1	1	0	0	0	4	1	0	0	2
M3+wife join	81 s	110 s	1	0	1	1	0	0	0	0	6	1	0	0	4
Boggle cont.	111 s	120 s	1	0	1	1	1	0	0	0	6	1	0	0	2
M2 joins	121 s	155 s	0	0	1	1	0	0	0	0	6	1	0	0	3
F4 joins	156 s	177 s	0	0	1	0	0	0	0	0	6	1	0	0	2
Boggle, set, go!	179 s	1739 s	1	1	1	1	1	0	1	1	7	1	0	0	5
Sign off	1740 s	1763 s	1	1	0	0	1	0	0	1	8	1	0	0	5

Abbreviations: Disc., disclosure; F, female participant; M, male participant; NVerb, non‐verbal.

For each event, we coded eight binary variables indicating whether a given behavior occurred. Based on the domain expertise of our team's researchers, the following behavioral codes were predicted to be relevant and were recorded for each event: social, meaning that the event consisted of some social interaction in addition to the game question/answer; humor, meaning that the event involved laughter or a joke of some kind; game question/answer, meaning that the event consisted of participants being asked a game question or answering a question; host cuing, meaning the facilitator offered a hint or signaled participants to behave in some way; affirmation, meaning the host provided verbal or non‐verbal positive feedback consisting of encouraging words or utterances to participants; self‐disclosure, meaning the host disclosed some personal information about him or herself; host correction, meaning the host corrected participants’ answers in some way; and notable non‐verbal response, meaning that some kind of interesting non‐verbal reaction by a host or participant was visible in the video recording (e.g., somebody shaking their head, or looking very surprised).

In addition to coding for behaviors, numerical data were identified for each event, including the start and end times of each event, which were used to obtain the corresponding pitch and intensity data, and the number of people engaged during an event. Finally, for each event we identified the host by coding three binary variables, with a value of “1” for the first three variables identifying Hosts 1, 2, and 3, respectively, and all three variables equal to “0” identifying Host 4. The deidentified event data recorded by the two coders is publicly available in the form of Microsoft Excel files on Github.^23^

### 2.5 Measures of social engagement

The degree of participant engagement during each event was rated by two investigators on a Likert scale from highest (5) to lowest engagement (1) using behavioral codes created for this analysis. After viewing the recordings several times, investigators identified host behaviors that were followed by active participation in the games or discussions. To guide the coding process, a list of behavioral codes was developed by one investigator with corresponding defining characteristics. Rater agreement was reviewed, and inter‐rater differences were discussed.^24^ The engagement score datasets for both raters are publicly available and can be found in the Microsoft Excel files containing the social event coding data.^23^

An iterative process occurred in which codes were refined and a few were collapsed (e.g., initially distinct forms of positive feedback were aggregated into affirmation), followed by both investigators rescoring overall engagement for all events.^25^, ^26^ Inter‐rater reliability was examined using percent agreement, where it was observed that 54.6% of all ratings agreed exactly while 38.5% of scores agreed within one point, resulting in 93.1% overall approximate agreement (see Figure S1 in supporting information for more details). Engagement scores were used as the output in multivariate linear regression models, with the predictors being behavioral variables, the number of people engaged, the host ID, and the acoustic–prosodic entrainment and time data described in the following subsections. Statistical significance of variables, proportion of variance explained, and coefficient size were used to determine the relative importance of predictors.^27^ Additionally, the engagement scores were compared as a function of host ID.

### 2.6 Acoustic data preparation and analyses

Audio–video recordings of the virtual group sessions were obtained, and audio data were extracted from the video files and converted from stereo to mono. Professional acoustic analysis software Praat^28^ was used to extract the corresponding pitch and intensity tiers, and Praat's annotation features were used to manually diarize the audio data, identifying who spoke when with markers H (host), C (control), F1, …, F9 (female participant 1, etc.), and M1, …, M7 (male participant 1, etc.). In addition, audio segments corresponding to noise, such as background and technical sounds, and significant over‐speak were marked as N and SO, respectively, and were excluded from the analysis. Overall, ≈ 140 MB of pitch tier data, ≈ 500 MB of intensity tier data, and ≈ 20 MB of diarization data were extracted from the 30 videos, all of which have been made publicly available.^23^

The distributions of pitch and intensity for each individual speaker were normalized for each video. This was done by converting each pitch and intensity value to a *Z* score by subtracting the mean and dividing by the standard deviation of the data for each speaker calculated over each video (see Figure S2 in supporting information for more details). Statistical analyses of these audio data were then used to test for a relationship between acoustic–prosodic entrainment^14^, ^15^, ^16^ and enhanced social engagement.^29^, ^30^, ^31^ We tested for acoustic proximity by computing the absolute differences in acoustic features for each sequential pair of turns, and averaging over all such pairs of turns in an event to obtain a single number for each event, using as features the mean pitch, maximum pitch, minimum pitch, mean intensity, maximum intensity, and minimum intensity, where the mean, maximum, and minimum are taken over each speaker's turn. We then used these averaged feature differences as predictors in linear regression models. Not all events had pairs of speakers, and hence 258 events which only had one speaker were excluded from the analysis, leaving a total of 1581 available for these analyses (for examples of other tests of acoustic–prosodic entrainment using our data, see Tables S1–S3 in supporting information).

### 2.7 Time data analyses

**FIGURE 1:**
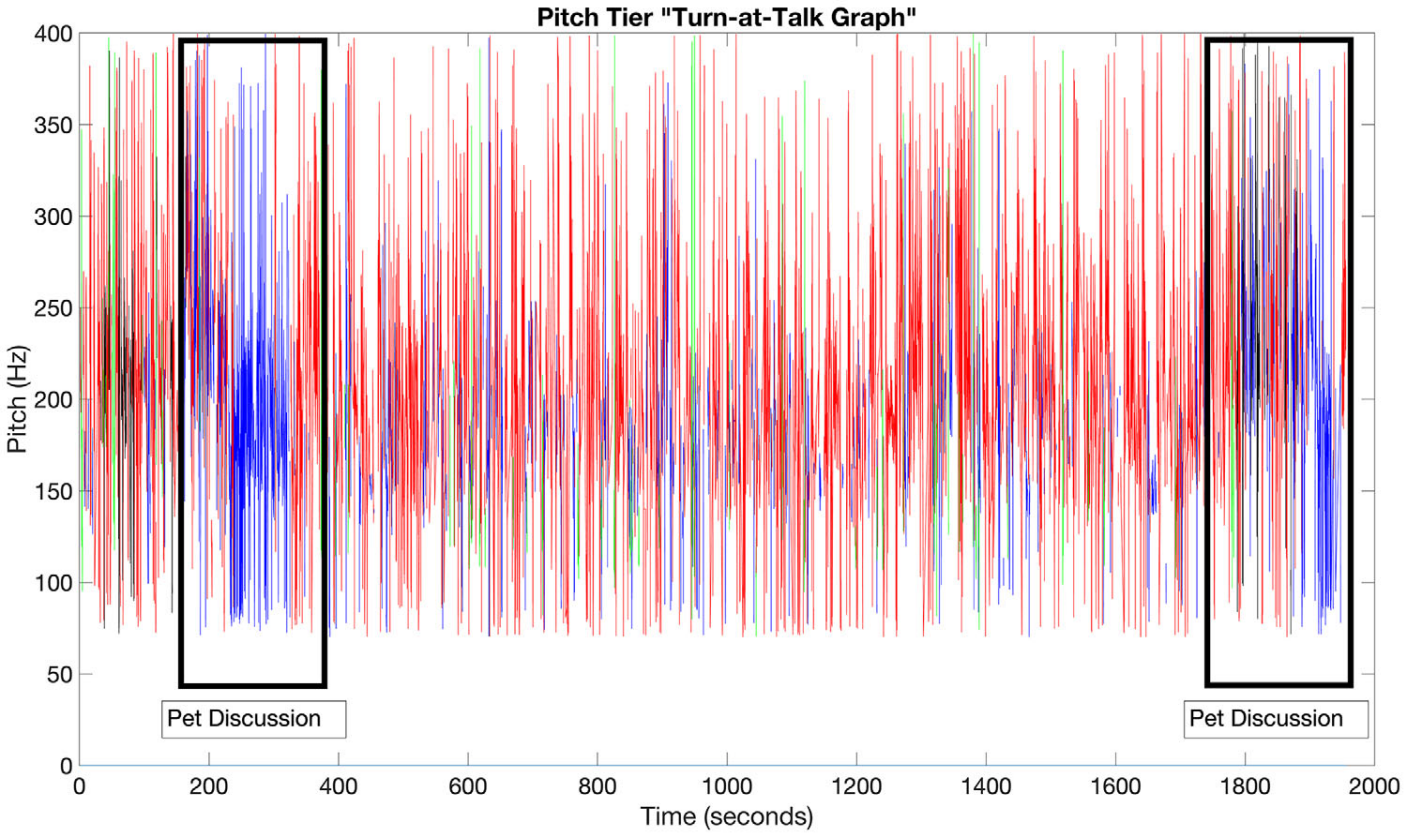
A “Turn‐at‐Talk” graph, illustrating turn segments and fluctuations in speaker pitch over time. Red identifies the host, blue identifies female participants, green identifies male participants, and black identifies the younger control subject. Note the unusually long blue segments that have been highlighted, which show female participants engaged in spontaneous, prolonged discussions about pets in response to the control subject presenting her pet cat to the camera on two different occasions.

From the audio signals we extracted time data for each event, including the amount of time each speaker group (host, control, participant) spoke, the percentage of time spent speaking by the participants, and the percentage of time that a video's audio data consisted of silence.^31^ We used these time data in multivariate regression analyses with the engagement scores as the response. We also examined global trends in time data by observing the percentage of time spoken by participants over entire videos and the distribution of the lengths of participant turns over all videos, which were compared to the local content of the videos using color‐coded “Turn‐at‐Talk Graphs” identifying the different speakers (see Figure 1). To reiterate section 2.2, ≈ 900 minutes or 15 hours worth of time data was collected for analysis.

## 3 RESULTS

### 3.1 Specific games and stimuli promote engagement

Exploratory analyses of global and local time‐based data identified multiple instances of spontaneous, prolonged conversations that were qualitatively different than any of the other interactions. These interactions were found to be elicited by specific conversational topics or stimuli that were identified by observing the corresponding audio–video data and using Turn‐At‐Talk Graphs. These included the visual displaying of the younger control subject's pet cat to the camera and discussions involving pets, discussions involving opinions on court cases, and singing the missing lyrics to songs.

Although the sample size of these types of interactions is very small (approximately a dozen instances), these topics resulted in the participants speaking far longer than any of the other data (percentile approximately equal to 100), and even resulted in multiple such very long turns when the topics were brought up at different times, suggesting that it was specifically these topics and stimuli that enhanced participant engagement (see Figure 1). Importantly, there was no direction from the host to speak longer than usual, supporting the conclusion that these specific stimuli and topics motivated the participants to spontaneously speak longer. Additionally, it was found that certain types of games produced more social engagement as measured by the percentage of time spoken by participants per video. Specifically, we found that improvised games with more egalitarian player roles, such as Boggle, resulted in a higher percentage of total time spent spoken by participants (> 40%), compared to other videos that were much more skewed.

### 3.2 Influence of host on engagement

**FIGURE 2:**
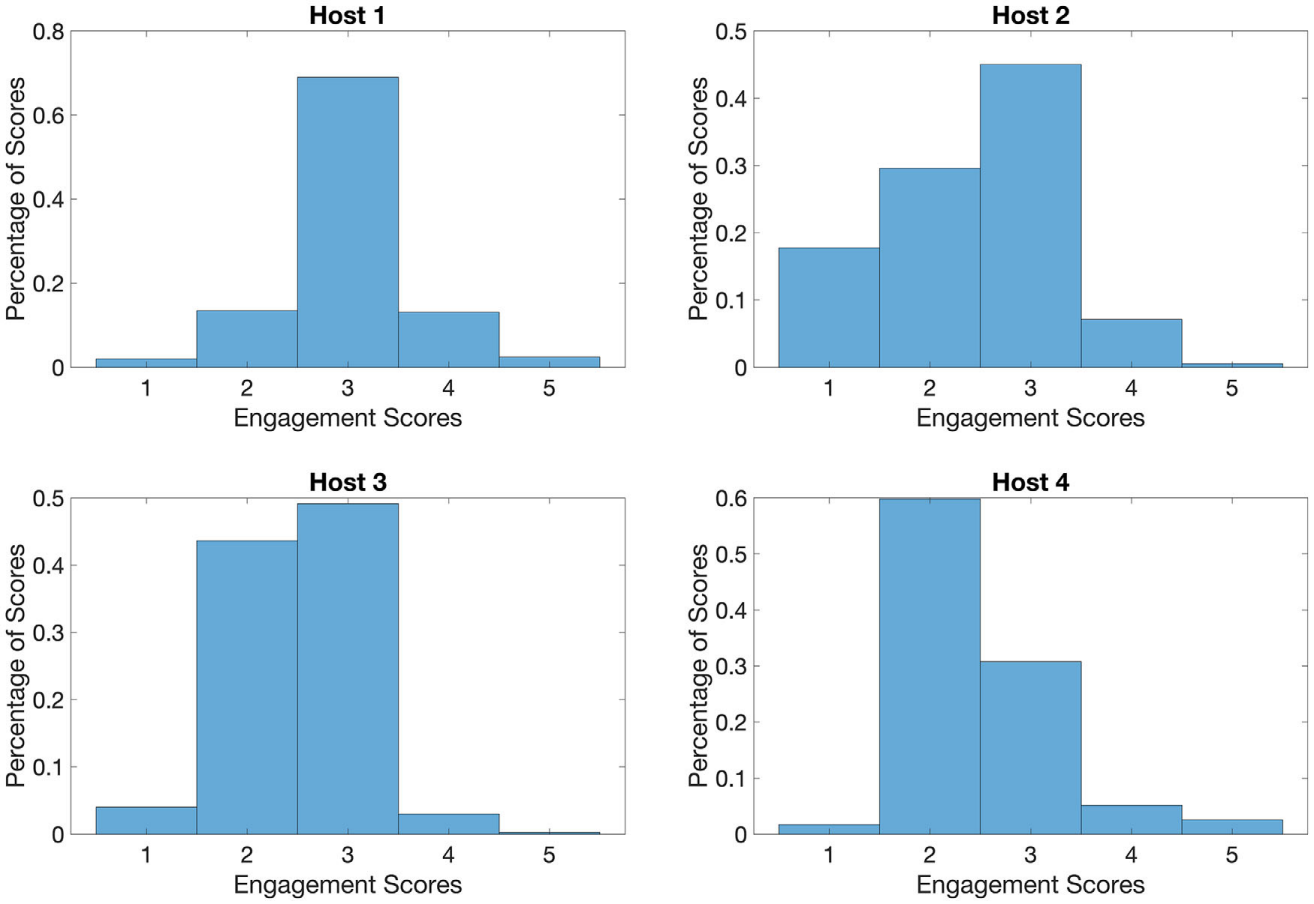
Histograms of the engagement scores for the four different hosts. Note that distribution of Host 1's scores resemble a true normal distribution, whereas the distributions for the remaining hosts are skewed negatively.

An exploratory analysis of the engagement scores identified significant differences in the levels of engagement for the different hosts. Figure 2 shows the distributions of engagement scores for the four hosts. Host 1's engagement scores tended to be higher than the others’, which tended to skew negative rather than resembling true normal distributions. These apparent differences in the engagement score distributions were confirmed by two statistical one‐way analysis of variance tests, one comparing Hosts 1, 2, 3, and 4 for 117 events, and another comparing just Hosts 1, 2, and 3 for 399 events. We down‐sampled the number of engagement data points to whichever host had the smallest number of events (Host 4 had 117 events, Host 3 had 399, Host 1 had 748 events, and Host 2 had 575). We found Host ID had moderate and large effect sizes (η^2^ = 0.0802 and η^2^ = 0.1285 for the two comparisons described above, respectively) with high statistical significance (*P* = 1.482e‐06 and *P* = 1.0462e‐22, respectively). Post hoc Tukey honestly significant difference tests for both comparisons confirmed that the mean of Host 1 was significantly different from the means of the other hosts.

The significance of host ID as a predictive variable for the engagement score was confirmed by incorporating host ID into linear regression models with the engagement score as the response (see Table 2). Host ID was found to be highly statistically significant with a very low *P* value (*P* = 2.36e‐48) and explained a moderate amount of variance (*R* ^2^ = 0.115). In addition, when considering each host ID individually as predictors, the coefficient for Host 1 was positive while the coefficients for Hosts 2 and 3 were negative, supporting the hypothesis that interactions with Host 1 tended to enhance engagement while interactions with the other hosts tended to reduce engagement (see Table S4 in supporting information for the corresponding regression model analyses for the second rater's set of engagement scores).

**TABLE 2:** Statistical data produced from fitting Host ID as predictor variables in linear regression models with the engagement score as the response.

Model data	Host 1	Host 2	Host 3
Individual coefficient	0.53655	−0.37012	−0.21315
Individual SE	0.03507	0.038501	0.044099
Individual *R* ^2^	0.113	0.0479	0.0126
Individual *P* value	7.943e‐50	2.2191e‐21	1.4538e‐06
Total coefficient	0.53393	−0.038781	0.048712
Total SE	0.07342	0.074899	0.077642
Total *R* ^2^	0.115	0.115	0.115
Total *P* value	5.2136e‐13	0.60468	0.53048

### 3.3 Influence of group size and the control participant

We found that the number of people speaking in each event was the single most significant predictor when used in linear regression models out of any of the predictors examined in this study, both alone and when included with the other predictors, explaining a large amount of variance alone (*R* ^2^ = 0.236) and having extremely high statistical significance (*P* = 2.22e‐109). Further, we found that the presence of and interaction with the younger control enhanced social engagement. This was determined with an exploratory analysis, in which it was observed that a friendly relationship between one of the male participants and the female control formed over the course of the study. This finding was confirmed by observing the change in the male participant's behavior during the exact same game played on two adjacent sessions, one when the control was present and the other without the control. During the session with the control, the participant spoke ≈ 30% of the time, versus < 3% in the subsequent session.

### 3.4 Other behaviors affecting engagement

The behavioral variables described in section 2.4 were incorporated into linear regression models predicting the engagement scores as outputs for each event, with the results displayed in Table 3. These variables were found to be highly statistically significant and together accounted for a moderate amount of variance (*P* value = 1.28e‐67; *R* ^2^ = 0.167). Humor, social, and non‐verbal response were found to be the most significant predictors of social engagement compared to other behavioral variables, having the largest coefficients, smallest *P* values, and most variance explained (see Table S5 in supporting information for the corresponding regression model analyses for the second rater's set of engagement scores).

**TABLE 3:** Statistical data produced from fitting the eight behavioral variables as predictors in linear regression models with the engagement score as the response.

Model data	Social	Humor	Game	Cuing	Affirm	Disclose	Correct	NVerbal
Individual coefficient	0.3713	0.58722	0.16021	0.19825	0.23488	0.34236	0.30256	0.7817
Individual SE	0.040007	0.048305	0.084703	0.036474	0.044202	0.055464	0.04554	0.1068
Individual *R* ^2^	0.0448	0.0745	0.00194	0.0158	0.0151	0.0203	0.0235	0.0283
Individual *P* value	4.57e‐20	8.1e‐33	0.0587	6.2e‐08	1.2e‐07	8.24e‐10	4.02e‐11	3.71e‐13
Total coefficient	0.25448	0.40899	0.20315	0.16597	0.29102	0.1096	0.21913	0.73436
Total SE	0.043239	0.050614	0.083607	0.034833	0.042383	0.054509	0.042992	0.10004
Total *R* ^2^	0.167	0.167	0.167	0.167	0.167	0.167	0.167	0.167
Total *P* value	4.71e‐09	1.15e‐15	0.0152	2.03e‐06	8.98e‐12	0.0445	3.81e‐07	3.18e‐13

Abbreviations: NVerbal, non‐verbal; SE, standard error.

### 3.5 Acoustic–prosodic entrainment and social engagement

The results of fitting the acoustic–prosodic entrainment data described in section 2.6 as predictors in linear regression models with the engagement scores as the response are shown in Table 4. These acoustic entrainment data together accounted for a very small amount of variance (*R* ^2^ = 0.0168) with a statistically significant fit (*p* = 0.00017), with Mean Pitch and Min Intensity individually explaining the most variance (*R* ^2^ = 0.0102 and *R* ^2^ = 0.0117, respectively). However, in the total acoustic model incorporating all variables, the statistical significance of all but Mean Pitch and Min Intensity was lost. This suggests either that acoustic–prosodic entrainment is a relatively insignificant predictor of social engagement, or that the specific entrainment measures used in our analysis are not sensitive to such engagement (see Table S6 in supporting information for the corresponding regression model analyses for the second rater's set of engagement scores).

**TABLE 4:** Statistical data produced from fitting the six acoustic–prosodic entrainment variables as predictors in linear regression models with the engagement score as the response.

Model data	Mean pitch	Max pitch	Min pitch	Mean intensity	Max intensity	Min intensity
Individual coefficient	−0.3245	−0.07522	−0.1009	−0.3158	−0.2675	−0.1843
Individual SE	0.080559	0.026341	0.042041	0.13198	0.081283	0.042658
Individual *R* ^2^	0.0102	0.00514	0.00363	0.00361	0.00682	0.0117
Individual *P* value	5.89e‐05	0.00435	0.0165	0.0168	0.00102	1.65e‐05
Total coefficient	−0.2313	0.005860	0.01713	0.1659	−0.1206	−0.1474
Total SE	0.10449	0.035426	0.050446	0.17679	0.11252	0.052116
Total *R* ^2^	0.0168	0.0168	0.0168	0.0168	0.0168	0.0168
Total *P* value	0.026941	0.86863	0.73414	0.34814	0.28411	0.00474

Abbreviation: SE, standard error.

### 3.6 Participant time and social engagement

The results of fitting the time data described in section 2.7 as features in linear regression models with the engagement scores as the response are shown in Table 5. We found that the time data alone accounted for a moderate amount variance (*R* ^2^ ≈ 0.10) with high statistical significance (*P* = 3.16e‐40), with the amount and percentage of time spoken by the participants having the smallest *P* values and highest *R* ^2^ values (see Table S7 in supporting information for the corresponding regression model analyses for the second rater's set of engagement scores).

**TABLE 5:** Statistical data produced from fitting the five time‐data variables as predictors in linear regression models with the engagement score as the response.

Model data	Host time	Control time	Participant time	Participant fraction	Silence fraction
Individual coefficient	0.0026551	0.060117	0.014438	1.7548	−0.23427
Individual SE	0.0005094	0.01586	0.0014056	0.15468	0.064233
Individual *R* ^2^	0.0146	0.00776	0.0543	0.0655	0.00719
Individual *P* value	2.08e‐07	0.000155	4.17e‐24	7e‐29	0.000272
Total coefficient	−0.00050297	0.024067	0.011943	1.39	−0.12233
Total SE	0.00075584	0.015592	0.0021627	0.16763	0.065851
Total *R* ^2^	0.101	0.101	0.101	0.101	0.101
Total *P* value	0.50585	0.12286	3.8298e‐08	2.1252e‐16	0.06338

Abbreviation: SE, standard error.

### 3.7 General social engagement model

After considering subsets of individual predictors and categories of predictors, we combined all the predictors into one multivariate regression model. We excluded the acoustic variables from this general model as we have shown them in section 3.5 to be relatively insignificant. The results of evaluating this model on the data for all 30 videos are given in Table 6. This model explained a high proportion of variance (*R* ^2^ = 0.397) and had very high statistical significance (*P* = 1.27e‐185, *F* statistic vs. constant model = 70.5). Further, we found that most predictors have statistically significant non‐zero coefficients, suggesting that they describe significant contributions to the overall engagement score (see Table S8 for the corresponding regression model analyses for the second rater's set of engagement scores).

**TABLE 6:** The results of fitting a general linear regression model using all previous predictors (except for the acoustic variables, which were determined to be relatively insignificant in the previous subsection) to all 1839 events (error degrees of freedom 1821).

Model parameter	Estimated coefficient	SE	*t* statistic	*p* value
(Intercept)	1.131	0.10919	10.359	1.7978e‐24
Social	0.1989	0.037473	5.3078	1.2456e‐07
Humor	0.19644	0.044217	4.4427	9.4162e‐06
Game	0.10563	0.073348	1.4401	0.15
Cuing	0.16058	0.03115	5.155	2.8118e‐07
Affirm	0.34932	0.036589	9.547	4.1257e‐21
Disclose	0.1291	0.047488	2.7186	0.006618
Correct	0.18716	0.037355	5.0102	5.9645e‐07
Non‐verbal	0.29145	0.087947	3.3139	0.00093803
Host 1	0.42914	0.065078	6.5943	5.5801e‐11
Host 2	0.040262	0.067352	0.59779	0.55006
Host 3	0.20137	0.069579	2.8941	0.0038479
Number	0.23704	0.014865	15.946	1.0978e‐53
Participant time	0.0057877	0.0018093	3.1988	0.0014036
Host time	−0.00078371	0.00064548	−1.2142	0.22484
Control time	0.035459	0.01315	2.6966	0.0070702
Participant fraction	0.60998	0.14563	4.1885	2.9425e‐05
Silence fraction	−0.060426	0.057182	−1.0567	0.29078

Abbreviation: SE, standard error.

## 4 DISCUSSION

Social engagement in later life requires sufficient sensory function to clearly perceive social partners, adequate memory to maintain conversation, executive skills to plan and time verbal responses, empathy, and self‐regulation to inhibit irrelevant responses or prolonged silence, yet these abilities are significantly impacted by ADRD and worsen with disease progression.^32^ Promoting and preserving robust engagement in groups of older adults with ADRD requires an understanding of the factors that influence engagement. Our work identified predictors of enhanced engagement in groups of older adults with ADRD interacting with younger adult facilitators, the most significant being the number of participants engaged. Complexity science predicts that complex systems with many interacting diverse subsystems have richer dynamics.^10^, ^11^, ^12^ This suggests that groups involving many participants with a range of cognitive abilities may produce higher engagement by promoting more complex interactions.

Although some participants in our study spoke very little, when they did speak, they typically offered meaningful responses relevant to the discussion, suggesting that they were not disengaged but rather simply spoke less. Limitations in language occur early in ADRD but loss of some abilities does not rule out social engagement in a group setting, given carefully planned support. This is supported by McCullough et al.^33^ who found that older adults with mild cognitive impairment (MCI) had lower linguistic scores compared to others with MCI and to their own performance on non‐language tests (e.g., visuospatial construction). Also, because the social task of interacting with many members in a group may be cognitively demanding for older individuals with ADRD, an intermediate group size may be best to maximize engagement.

We also identified behaviors associated with enhanced engagement, including use of humor, cuing, affirmation, and so forth, and considered the impact of other features on engagement including time‐based data and measures of acoustic–prosodic entrainment. We found essentially no connection between acoustic–prosodic entrainment and social engagement, contrasting with previous studies which had found a relationship between the two.^29^, ^30^, ^31^ This discrepancy may have many explanations: our use of virtual socialization software rather than microphones recording in‐person interactions, using measures of acoustic–prosodic entrainment that are not sensitive enough to detect correlations with engagement, or perhaps acoustic–prosodic entrainment is simply not significant in groups of older adults with ADRD.

Further, we found significant differences in the distributions of engagement scores for the different hosts, where the host with the highest overall engagement scores also had the most training and experience in working with the participant population. Additionally, third‐party stimuli, such as singing or the visual display of pets, significantly increased engagement. We also observed that participants became more comfortable speaking over time as the sessions progressed. This suggests that rapport developed, enabling participants to express themselves more freely over the multiple sessions.

### 4.1 Study strengths and limitations

A major strength of our study was the large amount of data collected. This includes 1839 social event datapoints, each with 17 features and 2*1839 = 3678 engagement scores, as well as 140 MB of pitch data, 500 MB of intensity data, and 20 MB of diarization data spanning 30 videos for a total of 900 minutes of time data collected.^23^ Our collection of high‐volume data quantifying social interactions and use of statistical modeling to optimize group dynamics builds on growing research trends in social science which are becoming increasingly common due to advances in data collection methods.^18^ However, our data are unique in that they derive from groups of older adults with ADRD.

We noted several limitations over the course of this study. The COVID‐19 pandemic began soon after this study was approved, forcing us to adapt the design. The MWC chose the video streaming software WebEx, which at the time did not provide automatic diarization of the speakers, and manual diarization was extremely time consuming. Also, the use of video streaming software introduces technical artifacts in the video data, including transmission delays on the order of a few milliseconds and audio filtering, which may have affected the data analysis. Finally, because our study site was closed during the period of data collection due to the COVID‐19–related suspension of in‐person activities, we were unable to extract comprehensive participant sociodemographic data from MWC records such as age, sex, gender, level of education, income, and ethnicity. We relied on MWC policies for attending the day center (e.g., older adults diagnosed with mild to moderate cognitive impairment due to ADRD) to describe the group.

### 4.2 Recommendations for application in clinical settings

Our study conducted a systemic, whole‐group analysis of social interactions and revealed that facilitators have the potential to improve engagement through a variety of strategies. To develop a successful group program promoting engagement for older adults with ADRD, we recommend that experts consider group composition, structure, leadership strategies, content, and mode of delivery.

Ideally, groups would consist of several older adult participants with a range of cognitive abilities along with younger adults to help support conversation and avoid long silences. To be included, older adults would have adequate hearing and receptive/expressive language ability for conversation. Cognitive reserve theory and complexity science support how a younger participant who joined the discussion intermittently enhanced engagement despite having weaker ties with regular members. She brought novel ideas, experiences, and emotional responses that enriched the social environment of the group. Familiar MWC attendees and hosts produced supportive but predictable responses. Both types of social interactions (bonding with close friends and relations and loose social connections with novel others) are important and support cognitive function in older adults.

Higher levels of engagement were observed in larger groups; however, the precise impact of group size on engagement needs further study in this population. Larger group size combined with stimulating interactions have been shown to increase cognitive reserve and protect against the threat of neurodegenerative disorders.^34^ Facilitators were found to have significant influence over participants’ level of engagement and that the extent of group leadership experience mattered. Hence, we recommend that leaders have a strong background in ADRD‐related strengths and deficits in receptive and expressive language, emotional perception, and processing, as well as group process. Facilitator training in engagement strategies such as the use of humor, cuing, and questioning would improve the likelihood of success.

Discussion of controversial topics such as court cases resulted in high levels of participant speech, perhaps because opinions were always well received and never judged right or wrong. Interactive games that were relevant to the life experience of participants, such as the music of their era, were more likely to elicit interaction. Topics need to be matched to participants’ age, interests, and sociodemographic characteristics such as level of education. Additionally, pets can be useful in aiding the health outcomes of older adults with and without ADRD,^35^, ^36^ and our results suggests that these benefits may even extend to virtual interactions. Either virtual or in‐person groups can be successful, but the advantages and disadvantages of each type may determine the optimal mode of delivery. For example, virtual groups accessed from home enlarge the potential pool of participants but may require involvement by a friend, family member, or home health aide to help the older adult use the technology. In‐person groups are limited by access to specialized care and staff availability.

### 4.3 Future research

Future research should consider the potential differences between in‐person and virtual socialization and attempt to identify and weigh the significance of other data that may be predictive of engagement. Additionally, follow‐up questioning of the older adults involved in such studies would be useful for determining the long‐term health benefits of these socialization programs. Virtual socialization combined with supportive digital technology for cognitively impaired older adults needs further development to expand access.^37^, ^38^ Finally, our negative results regarding the impact of acoustic–prosodic entrainment on engagement may be useful to future researchers in motivating which intervention techniques they choose to study.

### 4.4 Conclusion

ADRD is a prevalent health problem that affects millions of older adults around the world, with social isolation being among the most significant contributing factors. Isolation is growing among older adults and society in general. There is a bidirectional relationship between isolation and cognitive decline; those who are isolated are at higher risk for ADRD and those with ADRD are at higher risk of isolation due to lack of opportunity for contact, feelings of loneliness, worsening language skills, and social exclusion.

Our work demonstrates that virtual socialization software is a promising tool for mitigating isolation and identifies evidence‐based strategies that group facilitators can use to maximize engagement in social interactions. This provides isolated individuals opportunities for enriching socialization and combatting loneliness, which is a key contributor to the development and worsening of ADRD. Concepts from complexity science, such as the impact of whole‐group effects on individuals, third‐party stimulation, and measures of coordination, give insight into the mechanisms underlying the preservation and loss of social engagement, which can be used to reduce isolation and improve the health of older adults with ADRD.

## CONFLICT OF INTEREST STATEMENT

The authors declared no potential conflicts of interest with respect to the research, authorship, and/or publication of this article. Author disclosures are available in the supporting information.

## ETHICAL APPROVAL AND INFORMED CONSENT STATEMENTS

All research was conducted with approval of the Florida Atlantic University Institutional Review Board. Memory and Wellness Center family members consented while enrollees assented to taking part in the study.

## Supporting information



Supporting Information

Supporting Information

## Data Availability

All deidentified data used for this study, including the Praat‐extracted pitch and intensity tiers, the diarization logs, Excel spreadsheet event coding data, and MATLAB programs for running the analyses are publicly available on the Github data repository https://github.com/JosephCMcKinley/Enhancing‐Social‐Engagement.
