# Supplementary material for: Evidence‐based facilitator strategies for enhancing social engagement in groups of older adults with ADRD
Source: Alzheimers Dement. 2025 Apr 10;21(4):e70131. doi: 10.1002/alz.70131 (PMC11982927; doi:10.1002/alz.70131)
Supplement: Supplementary file 1 — Supporting Information [file ALZ-21-e70131-s001.docx]

**Evidence-based facilitator strategies for enhancing social engagement in groups of older adults with ADRD**

Supplemental Materials

Interrater Reliability

To determine the effects of various factors on the overall levels of social engagement, our team developed a system of subjective, professionally rated engagement scores. This scoring consisted of a process of identifying in the videos socialization events and assigning a Likert scale score of 1 (least engaging) to 5 (most engaging) for each event. Engagement scores were assigned to each event according to behavioral criteria, as well as the subjective judgment of the rater. Our study had two raters, one a geropsychiatric nursing scientist and the other a graduate research assistant. Interrater reliability (i.e., the consistency of the scores between the two raters) was evaluated using percent agreement. Percent agreement was determined by treating each Likert score as a categorical variable and constructing a normalized “confusion matrix”, where one rater’s scores were treated as the “true” scores and the other as the “predicted” scores (see Figure S1). This gives a complete description of the percent agreement, with the percentage of scores that agree exactly being given along the main diagonal and the scores that agree within one point adjacent to it. The percent agreement between the scores was generally high, with 54.1% exact agreement and 38.8% approximate agreement.


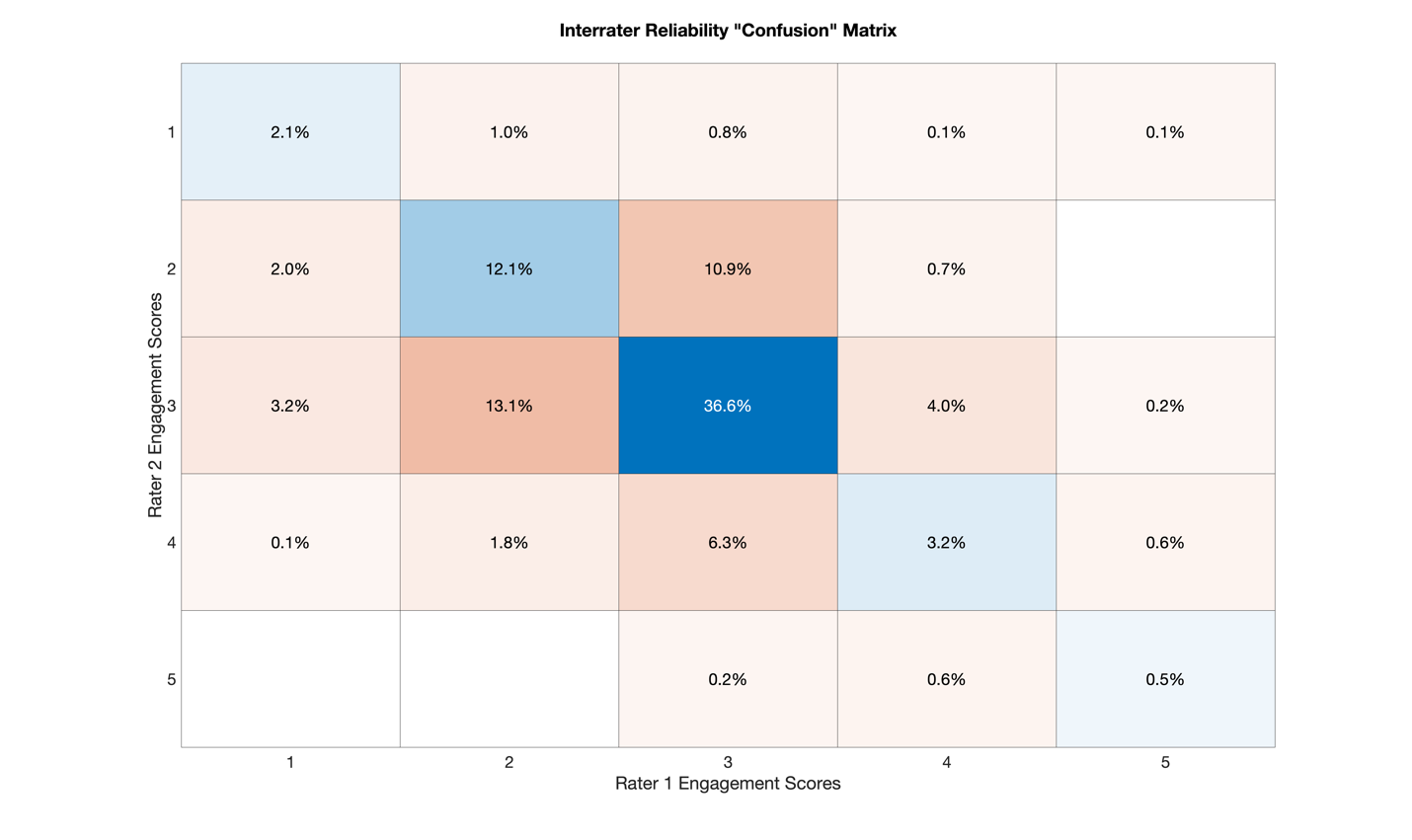


**FIGURE S1:** A “Confusion Matrix” displaying the degree of interrater agreement. Each box indicates the percentages of data consisting of that combination of ratings produced by the two raters, with the main diagonal giving the percentage of ratings that agree exactly and the boxes immediately adjacent to the main diagonal giving all the ratings that agree within one point. (Print in color)

Distributions of Acoustic-Prosodic Data

The data corresponding to acoustic-prosodic features (i.e., the pitch and intensity values for different speakers over time) were approximately normally distributed, with the mean and standard deviation unique to each speaker. Histograms corresponding to the pitch and intensity values for a single speaker over the course of a single video are shown in Figure S2. The shape of the data supports a normalization procedure whereby we convert each variable to a Z-score by subtracting the mean and dividing by the standard deviation for each individual speaker (where each statistic is computed over the course of a single video), setting the different distributions for different speakers all on the same scale.


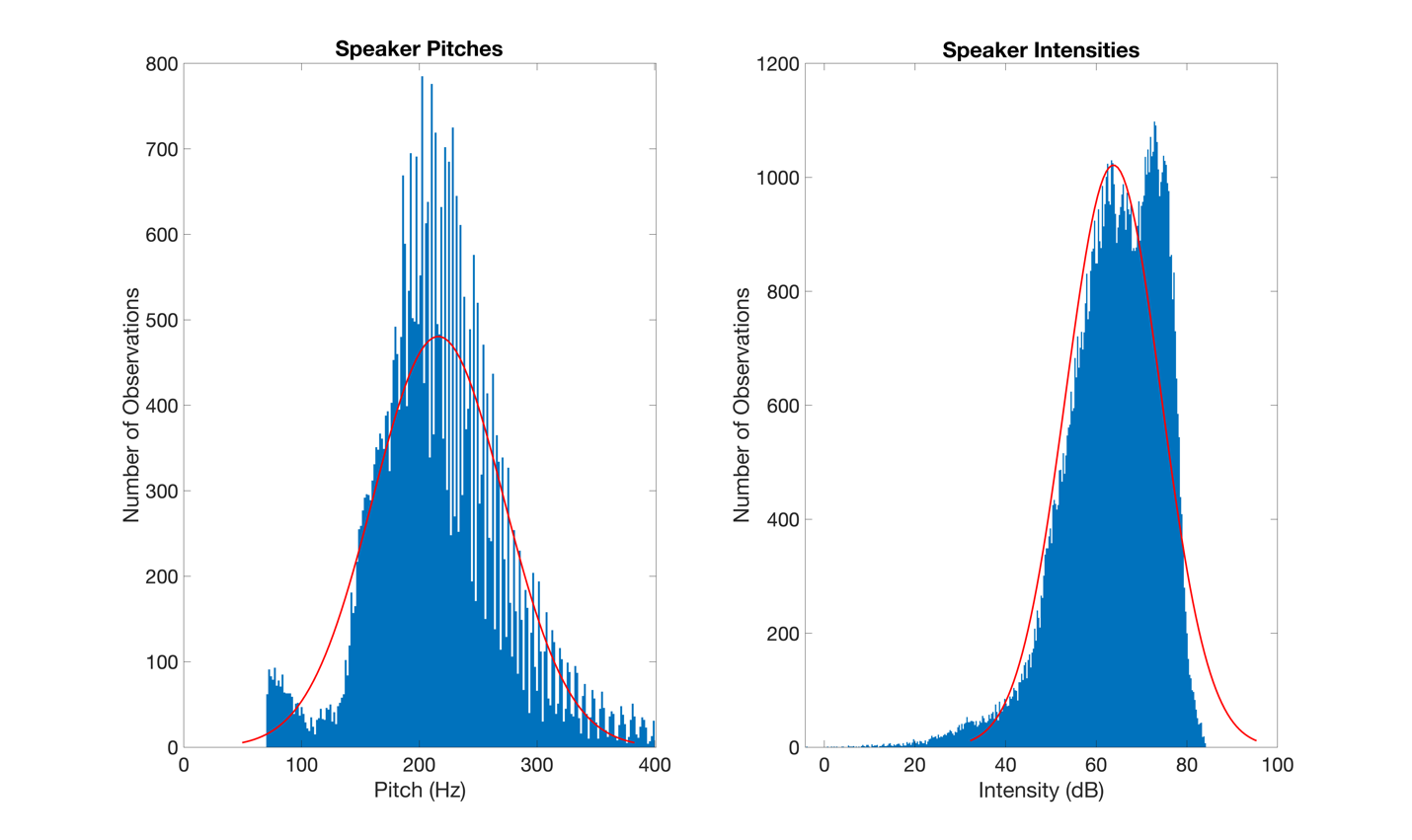


**FIGURE S2:** Histograms of the pitch and intensity values of speech segments for a single speaker taken over a single video. with a normal curve fit to each of them. This shape of the data supports a normalization scheme whereby each speaker pitch and intensity are converted to Z-scores.

Other Measures of Acoustic-Prosodic Entrainment

A significant body of research exists attempting to measure acoustic-prosodic entrainment during spoken interactions and its relationship to social engagement. One method, which inspired the acoustic-prosodic entrainment analysis conducted by our team, is that proposed by Levitan et al. whereby we employ statistical hypothesis tests to identify relationships between acoustic-prosodic features of different speakers. These tests include t-tests which measure acoustic *proximity* (i.e., the absolute closeness of acoustic statistics) by testing the hypothesis that acoustic features measured by conversational partners at turn exchanges have a smaller absolute difference than acoustic features measured away from turn exchanges. This is accomplished by compiling the acoustic features for all conversational partners at all turn exchanges for a given video and computing the absolute difference in those features, resulting in a vector of “partner differences” and performing a paired sample t-test where we test the hypothesis that the differences in those features at turn exchanges are smaller than the differences of the features of the first conversational partner in each turn and the average of 10 second speaker turns randomly selected from the video, where we refer to the latter vector of differences as a vector of “other differences”. This gives a t-score that measures acoustic proximity for each video.

A second statistical test proposed by Levitan et al. is one that measures for *synchrony*, which describes patterns in the acoustic-prosodic features of conversational partners over time in which partners tend to coordinate their features in response to one another. This can be measured by compiling the acoustic-prosodic features of all pairs of turns into two separate vectors for each turn in the pairs and computing the Pearson correlation between the two vectors. In addition to positive correlation coefficients, which suggest a form of in-phase synchronization (i.e., partners tend to move their pitches in step with each other), we note that the correlation coefficients can be negative as well, indicating anti-phase synchronization (i.e., one partner raises their pitch in response to the other lowering their pitch, and vice versa). Anti-phase synchronization is an important fundamental form of coordination that is ubiquitous in complex systems and allows for the emergence of complex spatiotemporal patterns of coordination between coordinative agents. We note that many instances of anti-phase synchrony (i.e., negative correlation coefficients) were observed for our videos.

Tables S1 and S2 give example results of the acoustic-prosodic proximity and synchrony tests for a single video, respectively. The features examined are the same statistics that are used in the main text: mean pitch, maximum pitch, minimum pitch, mean intensity, maximum intensity, and minimum intensity, where the statistics are taken over entire speaker turns. In Table S3, we give the results of these analyses applied to all 30 videos, with a “P” indicating a statistically significant result (p-value < 0.05) for proximity, and an “S” indicating a significant result for synchrony. The statistical results in Table S3 were then compared with the video data to see if they are predictive of a qualitative enhancement of engagement.  For instance, we might expect that a video with many statistically significant instances of acoustic proximity and synchrony would appear to be obviously “more engaging” than one with opposite statistics.

Statistically significant instances of proximity and synchrony were identified for many acoustic features over various videos. We found more instances of entrainment for pitch than intensity, with 30 such instances compared with intensity’s 15 instances. It’s worth noting that many of the observed synchrony coefficients were negative, suggesting coordination more akin to anti-phase synchrony. Although this analysis method did identify many instances of statistically significant acoustic-prosodic entrainment, it was not obvious how to interpret these results with respect to the video data. Reviewing the videos with the highest number of statistically significant results did not help our team identify factors related to enhanced engagement. Significant results, while interesting, did not serve our goal to identify methods for enhancing social engagement in older adults with ADRD or to evaluate the relative contribution of factors including acoustic-prosodic entrainment.

| **Feature** | **t-stat** | **Df** | **p-value** | **Sig.** |
| --- | --- | --- | --- | --- |
| Pitch Mean | -2.3208 | 508 | 0.02069 | Yes |
| Pitch Max | -1.3117 | 508 | 0.19019 | No |
| Pitch Min | 1.8443 | 508 | 0.06571 | No |
| Intensity Mean | 0.2175 | 508 | 0.82785 | No |
| Intensity Max | 0.4784 | 508 | 0.63254 | No |
| Intensity Min | 0.2606 | 508 | 0.79444 | No |

**TABLE S1:** Measures of acoustic-prosodic proximity as outlined in the previous subsection for a single video.

| **Feature** | **r** | **p-value** | **Sig.** |
| --- | --- | --- | --- |
| Pitch Mean | 0.11886 | 0.00726 | Yes |
| Pitch Max | 0.09051 | 0.04122 | Yes |
| Pitch Min | -0.08218 | 0.06390 | No |
| Intensity Mean | 0.01443 | 0.74522 | No |
| Intensity Max | -0.00798 | 0.85744 | No |
| Intensity Min | 0.06899 | 0.12001 | No |

**TABLE S2:** Measures of acoustic-prosodic synchrony as outlined in the previous subsection for a single video.

| **Video Date** | **Mean Pitch** | **Maximum Pitch** | **Minimum pitch** | **Mean Intensity** | **Maximum Intensity** | **Minimum Intensity** |
| --- | --- | --- | --- | --- | --- | --- |
| 04_09_2021 | S |  | S |  |  |  |
| 04_16_2021 | S |  | P, S |  | P, S |  |
| 04_21_2021 |  |  |  |  |  |  |
| 04_28_2021 |  |  |  | S |  |  |
| 05_03_2021 |  |  | P, S |  |  |  |
| 05_10_2021 |  | P, S |  |  | P, S |  |
| 05_14_2021 |  | P | P, S |  | P, S |  |
| 05_19_2021 | P, S | S |  |  |  |  |
| 05_21_2021 | P |  | P, S |  |  |  |
| 05_24_2021 |  |  | P, S |  |  |  |
| 06_04_2021 |  |  | P, S |  |  |  |
| 06_16_2021 | S | P | P, S | S | P |  |
| 06_21_2021 | S | P, S | P, S |  | P | S |
| 07_09_2021 |  | P |  |  |  | P, S |
| 07_14_2021 | P, S |  |  |  | S |  |
| 07_19_2021 |  | P, S | P, S |  | P, S |  |
| 07_23_2021 | S | P, S | S |  | P, S |  |
| 07_28_2021 | P, S | P |  |  |  |  |
| 07_30_2021 |  | P, S |  |  |  | P, S |
| 08_04_2021 |  | P, S | S |  | P, S |  |
| 08_09_2021 |  |  | P, S |  | P, S | P, S |
| 08_11_2021 | P, S | P, S | P, S |  | P |  |
| 08_20_2021 |  |  | P, S | S | P |  |
| 09_22_2021 | P, S | P |  |  |  |  |
| 10_01_2021 | P, S | P | P, S |  |  | P, S |
| 10_06_2021 |  | P, S |  |  |  |  |
| 10_29_2021 |  | P, S | P, S |  | P, S | P, S |
| 11_10_2021 | S | P, S | P, S |  | P, S | P, S |
| 11_12_2021 |  | P |  |  |  | P |
| 11_24_2021 |  | P, S |  |  |  | P |

**TABLE S3:** Table showing the results of the acoustic-prosodic entrainment analysis. A “P” indicates that the statistical test found statistically significant evidence of speech proximity, whereas a “S” indicates the same for synchrony (where statistical significance is defined by a p-value < 0.05).

Model Consistency Between Raters

To evaluate the consistency of the results of the regression models developed in the main text, we redid the analyses using the second rater’s engagement scores as the response (in the main text, we used the first rater’s scores). The results are shown in Tables S4-S8. We also performed ANOVA and Tukey post hoc HSD analyses for the different host engagement scores for the second rater as well, using the same scheme outlined in the main text (i.e., we do them for 117 randomly selected data points for hosts 1, 2, 3, and 4, and 339 such data points for hosts 1, 2, and 3). This identified large and moderate effect sizes, respectively (η^2^ = 0.153 and η^2^ = 0.0518) for the two cases.

| **Model Data** | **Host 1** | **Host 2** | **Host 3** |
| --- | --- | --- | --- |
| Individual Coefficient | 0.37539 | -0.13822 | -0.12294 |
| Individual SE | 0.034294 | 0.037366 | 0.042085 |
| Individual R^2^ | 0.0612 | 0.00739 | 0.00462 |
| Individual p-value | 4.6541e-27 | 0.000223 | 0.00353 |
| Total Coefficient | 0.85126 | 0.53356 | 0.53229 |
| Total SE | 0.070739 | 0.072164 | 0.074807 |
| Total R^2^ | 0.0903 | 0.0903 | 0.0903 |
| Total p-value | 5.39E-13 | 0.59972 | 0.55109 |

**TABLE S4:** Relevant data produced from fitting Host ID as a predictor variable in a linear regression model with the engagement score as the response (using the other rater’s engagement scores rather than those used in the main text).

As one can see by comparing Tables S4-S8 with Tables 2-6 in the main text, most of the main qualitative findings are retained across the two sets of engagement scores. The main difference is that most of the models fitted to the second rater’s engagement scores have a somewhat lower proportion of variance explained, while the behavioral variable model had an R^2^ = 0.0573 which was substantially smaller than the R^2^ = 0.167 in the main text. This can be explained by the fact that the rater scores used in the main text (Rater 1) were scored by a geropsychiatric nursing scientist with extensive prior experience with behavioral and social science. Further, this rater was primarily responsible for developing the behavioral codes used in the main text and likely paid closer attention to rating the scores according to these preestablished codes, whereas the scores used here (Rater 2) were rated by a graduate research assistant who relied more on subjective judgment when rating the scores and who lacked the relevant background that Rater 1 had.

| **Model Data** | **Social** | **Humor** | **Game** | **Cuing** | **Affirm** | **Disclose** | **Correct** | **NVerbal** |
| --- | --- | --- | --- | --- | --- | --- | --- | --- |
| Individual Coefficient | 0.2581 | 0.37398 | -0.00540 | 0.00066397 | 0.096356 | 0.10149 | 0.026703 | 0.46964 |
| Individual SE | 0.038297 | 0.046566 | 0.080169 | 0.035163 | 0.043224 | 0.054059 | 0.054893 | 0.10335 |
| Individual R^2^ | 0.0241 | 0.0339 | 2.48e-06 | 1.94e-07 | 0.0027 | 0.00192 | 0.000129 | 0.0111 |
| Individual p-value | 2.12e-11 | 1.71e-15 | 0.946 | 0.985 | 0.0259 | 0.0606 | 0.627 | 5.87e-06 |
| Total Coefficient | 0.19993 | 0.28603 | 0.11201 | -0.0015709 | 0.13526 | -0.058873 | 0.014093 | 0.41939 |
| Total SE | 0.044011 | 0.051035 | 0.084388 | 0.035524 | 0.043948 | 0.056406 | 0.05449 | 0.10202 |
| Total R^2^ | 0.0573 | 0.0573 | 0.0573 | 0.0573 | 0.0573 | 0.0573 | 0.0573 | 0.0573 |
| Total p-value | 5.9194e-06 | 2.4038e-08 | 0.18457 | 0.96473 | 0.0021161 | 0.29675 | 0.79594 | 4.118e-05 |

**TABLE S5:** Relevant data produced from fitting the eight behavioral variables as predictors in linear regression models with the engagement score as the response (using Rater 2’s scores).

| **Model Data** | **Mean Pitch** | **Max Pitch** | **Min Pitch** | **Mean Intensity** | **Max Intensity** | **Min Intensity** |
| --- | --- | --- | --- | --- | --- | --- |
| Individual Coefficient | -0.12761 | -0.070714 | -0.05701 | -0.25136 | -0.16192 | -0.10765 |
| Individual SE | 0.077698 | 0.0253 | 0.040424 | 0.12683 | 0.078225 | 0.041121 |
| Individual R^2^ | 0.00171 | 0.00492 | 0.00126 | 0.00248 | 0.00271 | 0.00432 |
| Individual p-value | 0.101 | 0.00525 | 0.159 | 0.0477 | 0.0386 | 0.00893 |
| Total Coefficient | 0.018815 | -0.052356 | 0.012339 | -0.031035 | -0.019108 | -0.071755 |
| Total SE | 0.10087 | 0.034198 | 0.048697 | 0.17066 | 0.10862 | 0.050309 |
| Total R^2^ | 0.00663 | 0.00663 | 0.00663 | 0.00663 | 0.00663 | 0.00663 |
| Total p-value | 0.85205 | 0.12598 | 0.80001 | 0.85572 | 0.86038 | 0.15398 |

**TABLE S6:** Relevant data produced from fitting the six acoustic-prosodic entrainment variables as predictors in linear regression models with the engagement score as the response (Rater 2 scores).

| **Model Data** | **Host Time** | **Control Time** | **Participant Time** | **Participant Fraction** | **Silence Fraction** |
| --- | --- | --- | --- | --- | --- |
| Individual Coefficient | 0.0017181 | 0.076413 | 0.012121 | 1.2383 | -0.27242 |
| Individual SE | 0.00048611 | 0.015029 | 0.0013444 | 0.14932 | 0.060945 |
| Individual R^2^ | 0.00675 | 0.0139 | 0.0424 | 0.0361 | 0.0108 |
| Individual p-value | 0.000419 | 4.06e-07 | 4.77e-19 | 2.1e-16 | 8.3e-06 |
| Total Coefficient | -0.0023209 | 0.043943 | 0.013964 | 0.74362 | -0.24878 |
| Total SE | 0.00075587 | 0.015592 | 0.0021628 | 0.16764 | 0.065853 |
| Total R^2^ | 0.0792 | 0.0792 | 0.0792 | 0.0792 | 0.0792 |
| Total p-value | 0.001436 | 0.0034329 | 2.5485e-11 | 4.2737e-06 | 8.9049e-05 |

**TABLE S7:** Relevant data produced from fitting the five time-data variables identified as important predictors in linear regression models with the engagement score as the response (Rater 2 scores).

| **Model Parameter** | **Estimated Coefficient** | **SE** | **tStat** | **p-Value** |
| --- | --- | --- | --- | --- |
| (Intercept) | 1.2096 | 0.11455 | 10.559 | 2.4526e-25 |
| Social | 0.17032 | 0.039289 | 4.3352 | 1.536e-05 |
| Humor | 0.12799 | 0.045915 | 2.7875 | 0.0053673 |
| Game | 0.10184 | 0.076508 | 1.331 | 0.18334 |
| Cuing | -0.062976 | 0.032347 | -1.9469 | 0.051704 |
| Affirm | 0.20135 | 0.039193 | 5.1374 | 3.0845e-07 |
| Disclose | -0.06973 | 0.050725 | -1.3747 | 0.16941 |
| Correct | -0.080841 | 0.049229 | -1.6422 | 0.10073 |
| Nonverbal | 0.078151 | 0.092515 | 0.84474 | 0.39837 |
| Host 1 | 0.78809 | 0.0678892 | 11.608 | 4.1655e-30 |
| Host 2 | 0.67413 | 0.069663 | 9.677 | 1.2404e-21 |
| Host 3 | 0.61282 | 0.072572 | 8.4444 | 6.1534e-17 |
| Number | 0.21648 | 0.015638 | 13.843 | 1.6399e-41 |
| Participant Time | 0.0092306 | 0.0018848 | 4.8975 | 1.0565e-06 |
| Host Time | -0.0021169 | 0.00067479 | -3.1372 | 0.0017332 |
| Control Time | 0.04724 | 0.013766 | 3.4317 | 0.00061321 |
| Participant Fraction | 0.42435 | 0.15168 | 2.7976 | 0.0052027 |
| Silence Fraction | -0.078675 | 0.059658 | -1.3188 | 0.18742 |

**TABLE S8:** The results of fitting a general linear regression model using all previous predictors (except for the acoustic variables, which were determined to be relatively insignificant in the previous subsection) to all 1839 events (error degrees of freedom 1821, R^2^ = 0.276, p = 1.18e-114, F-statistic vs. constant model = 40.9).
